# Is Teledentistry as Effective as Clinical Dental Diagnosis in Pediatric Patients?

**DOI:** 10.1111/ipd.13290

**Published:** 2024-12-25

**Authors:** Müge Erbay Mola, Dilşah Çoğulu, Ece Eden, Aslı Topaloğlu

**Affiliations:** ^1^ Private Dentist Manisa Turkey; ^2^ Department of Pediatric Dentistry, Faculty of Dentistry Ege University İzmir Turkey; ^3^ Department of Pediatric Dentistry, Faculty of Dentistry Istanbul University‐Cerrahpasa İstanbul Turkey

**Keywords:** COVID‐19, diagnosis, pediatric dentistry, teledentistry

## Abstract

**Background:**

Teledentistry integrates telecommunications with dental practice, facilitating the exchange of clinical information and images for remote dental consultation and treatment planning. This approach enables dental care access across long distances, addressing the need for flexible healthcare solutions.

**Aim:**

This study aimed to evaluate the effectiveness of teledentistry compared to clinical in‐person dental diagnosis in pediatric patients during the COVID‐19 pandemic.

**Design:**

The study sample consisted of 200 children aged 3–13 years, each undergoing both teledentistry‐based and in‐person clinical dental diagnosis. Caries index scores, including DMFT/dmft and DMFS/dmfs, as well as the identification of specific dental conditions such as molar incisor hypomineralization (MIH), black tooth staining, periodontal disease, dental trauma, and orthodontic anomalies, were recorded in both diagnostic settings. Statistical analysis was conducted using chi‐square, Wilcoxon, and Fisher's Exact tests.

**Results:**

The mean age of participants was 7.86 ± 2.40 years. Caries index scores (DMFT/dmft, DMFS/dmfs) showed compatibility between teledentistry and clinical diagnoses. While “d/D, f/F, ds/DS, fs/FS” scores were observed to be higher in clinical diagnoses compared to teledentistry, the difference was not statistically significant (*p* > 0.05). Scores for “m/M” and “ms/MS” were identical in both diagnostic methods. Additionally, the prevalence of dental anomalies, including MIH, black tooth staining, periodontal disease, dental trauma, and orthodontic anomalies, was comparable across both diagnostic approaches.

**Conclusion:**

Findings suggest that teledentistry serves as an effective alternative to clinical in‐person diagnosis for pediatric dental consultations and treatment planning, demonstrating comparable accuracy in identifying caries and dental anomalies in children.

**Summary**

- Why this paper is important to paediatric dentists?
  ○ This study represents one of the most extensive comparative analyses between teledentistry and conventional clinical diagnosis within pediatric dentistry, providing valuable insights into its practical application and diagnostic accuracy.
  ○ Results affirm that teledentistry is a dependable tool for diagnosing dental conditions in pediatric patients, supporting its potential as an effective, alternative diagnostic approach.
  ○ The rapid expansion of teledentistry aligns with societal shifts driven by advancements in information and communication technologies and reduced operational costs, highlighting its increasing role in accessible and scalable dental care.

## 1 Introduction

Teledentistry integrates telecommunications technology and dental practice and facilitates remote dental consultation and treatment planning by transmitting clinical data and images over long distances [1, 2]. Recent advances in telecommunication technology have driven the development of specialized digital hardware and software tailored to patient screening, follow‐up, and diagnostic imaging, enhancing the capability of remote dental services [3, 4]. This innovative approach has proven particularly valuable in rural and remote areas, where access to specialist dental care often requires considerable travel, and for patients with limited mobility who may face challenges in accessing in‐clinic consultations [5, 6].

The COVID‐19 pandemic highlighted telemedicine's role in mitigating viral transmission among patients, families, and healthcare providers, with teledentistry becoming a critical tool in maintaining dental care access while adhering to social distancing protocols [7]. Health authorities worldwide have promoted telehealth to minimize direct contact between dentists and patients, helping to control the spread of COVID‐19 [8, 9, 10].

In pediatric dentistry, teledentistry facilitates early detection of potential oral health issues, allowing for timely interventions and providing personalized guidance on oral hygiene and preventive practices. This technology offers distinct advantages for children, particularly those in geographically isolated areas or from families with limited access to dental care. Through remote consultations, monitoring, and specific treatments (such as medication prescriptions, oral hygiene, and diet counseling), teledentistry allows timely interventions, reduces the necessity for in‐person visits, and can serve as a child's initial, low‐stress introduction to dental care within a familiar environment. By minimizing the need for travel and enabling preventive care through regular check‐ups and follow‐ups, teledentistry may also help reduce healthcare costs and prevent minor dental issues from escalating [11, 12].

Despite its promise, teledentistry in pediatric care faces several challenges that require attention to maximize its efficacy. Key concerns include ensuring patient privacy, safeguarding data security, and adhering to regulatory and licensing standards [13]. Patients must be informed of potential risks associated with misdiagnosis and treatment planning, which may arise due to technical limitations. Additionally, even with robust security measures like end‐to‐end encryption and two‐factor authentication, there remains a risk of data breaches during electronic information transmission, which should be transparently communicated to patients and families [14].

Previous studies on teledentistry have largely concentrated on adult cohorts or particular dental conditions, leaving a significant gap in extensive research assessing its reliability across diverse pediatric dental cases. This study seeks to bridge that gap by directly comparing the diagnostic accuracy of teledentistry and clinical in‐person dental diagnosis in pediatric patients. The focus on this demographic is essential, as pediatric patients present unique behavioral and diagnostic complexities that may impact the efficacy of remote diagnostic approaches. The present study aimed to evaluate the effectiveness of teledentistry compared to clinical in‐person dental diagnosis for pediatric patients during the COVID‐19 pandemic era.

## 2 Materials and Methods

### 2.1 Study Population and Ethical Approval

The study included 200 pediatric patients, aged 3–13 years, who presented to a private dental clinic for diagnostic evaluation during the COVID‐19 pandemic. Ethical approval was obtained from the Ethics Committee of Ege University Medical Faculty (Reference No: 21–2.1 T/62). Informed consent was initially obtained via phone from parents of the children requesting an appointment, with preliminary consent indicated by the parents' reply to a WhatsApp message stating, “I have read, understood, and approve.” Additionally, parents and patients aged 8–13 years signed formal consent forms during the in‐person clinical visit.

### 2.2 Inclusion Criteria

Participants were selected based on the following inclusion criteria:

- Pediatric patients and their parents who consented to participate.
- Cooperative patients, assessed through the availability of diagnostic images.
- Parents with active use of the “WhatsApp” application.

### 2.3 Diagnostic Protocol

Both teledentistry and clinical in‐person diagnoses were conducted by the same pediatric dentist (MEM) to maintain diagnostic consistency. Teledentistry diagnosis sessions were scheduled in consultation with the parents, involving an initial asynchronous exchange of intraoral photographs and videos, followed by a synchronous video consultation. To ensure consistency and minimize plaque‐related diagnostic discrepancies, parents were instructed to brush their children's teeth before capturing any images or videos.

### 2.4 Data Security and Documentation

Standard WhatsApp software with end‐to‐end encryption was used for secure data sharing and storage. Diagnostic questionnaires and forms were completed in real‐time during the teledentistry video session. All multimedia data, including photographs and videos, were securely stored on an encrypted online platform (Google Drive).

### 2.5 Data Collection Instruments and Diagnostic Criteria

The structured questionnaire and diagnostic form collected detailed information on the following parameters:

- *General Health Status*: Documented for each patient.
- *Caries Risk Assessment*: Evaluated using the Caries Risk Assessment Tool (CAT) [15].
- *Dental Caries Assessment*: DMFT/dmft and DMFS/dmfs indices were recorded according to WHO (World Health Organization) criteria [16].
- *Periodontal Status*: Classified as either healthy or unhealthy gingiva, with inflammation indicators such as redness, swelling, or bleeding.
- *Additional Conditions*: The presence of molar incisor hypomineralization (MIH), black tooth staining, dental trauma, and orthodontic anomalies were recorded as either present or absent.

### 2.6 Clinical in‐Person Diagnosis Protocol

**FIGURE 1:**
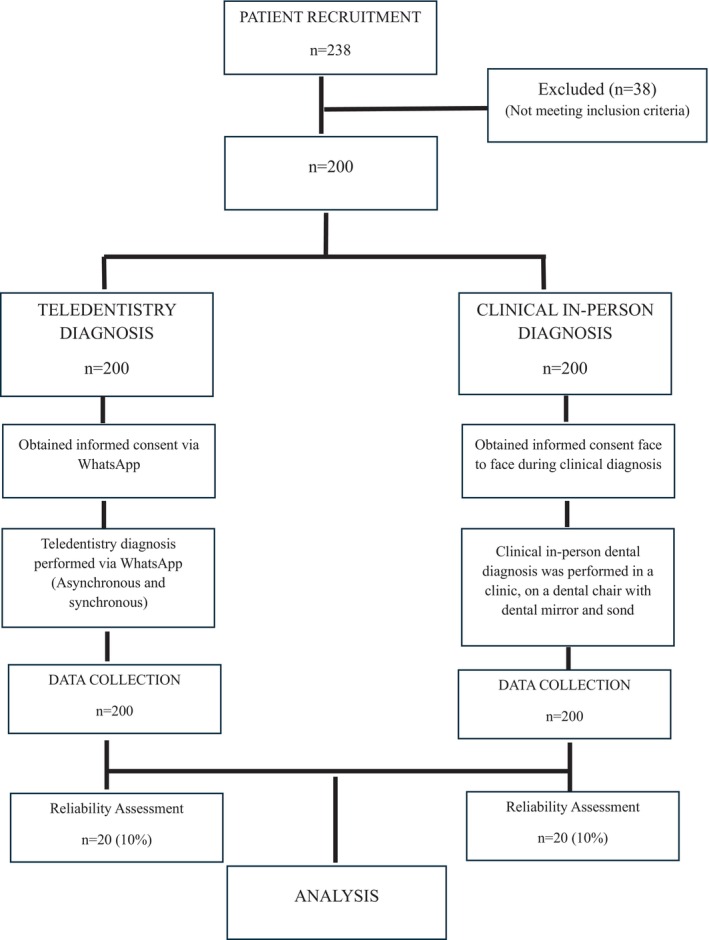
Flow chart summarizing the study process.

One week following the initial teledentistry evaluation, each patient attended a clinical diagnosis appointment. This in‐person assessment was conducted in a private dental clinic using a dental mirror and probe under reflector light, allowing for a comprehensive evaluation of dental health. Assessments included caries index scores (DMFT/dmft, DMFS/dmfs), identification of dental anomalies, and documentation of additional findings such as MIH, black tooth staining, periodontal status, dental trauma, and orthodontic anomalies. Figure 1 summarizes the teledentistry and clinical in‐person diagnosis processes.

### 2.7 Reliability Assessment

To evaluate the reliability of both teledentistry and clinical in‐person diagnostic methods, repeated evaluations were conducted. One week post‐initial assessment, the same examiner re‐evaluated a randomly selected subset of 20 patients (10% of the sample population). Intraclass correlation coefficients were calculated based on these repeat examinations to assess measurement consistency.

### 2.8 Statistical Analysis

Data analyses were performed using chi‐square, Wilcoxon, and Fisher's Exact tests within SPSS 22.0 software (SPSS Inc., Chicago, IL, USA). Statistical significance was determined at a 95% confidence interval with a threshold of *p* < 0.05.

## 3 Results

### 3.1 Patient Demographics

The cohort consisted of 200 children with a mean age of 7.86 ± 2.40 years, comprising 52.5% females (*n* = 105) and 47.5% males (*n* = 95). All patients who attended clinical in‐person examinations displayed compliant behavior (rated as 3 (positive) or 4 (definitely positive) on the Frankl Behavior Scale).

### 3.2 Caries Risk Assessment

Caries risk evaluation revealed by CAT that 23% (*n* = 46) of the patients were classified as low‐risk, while the remaining 77% (*n* = 154) exhibited high caries risk. Teledentistry assessments initially indicated 56 caries‐free patients; however, follow‐up clinical evaluations identified dental caries in 10 of these patients, reducing the clinically confirmed caries‐free rate to 23% (*n* = 46).

Mean dmft and dmfs scores (± SD) are displayed in Table 1. Clinically derived dmft and dmfs scores were generally higher than those determined through teledentistry for all age groups, except those aged 11–13. However, this variance did not achieve statistical significance (*p* > 0.05). Mean scores for individual indices (“d, m, f, ds, ms, and fs”) are presented in Tables 2 and 3. The “d, f, ds, and fs” parameters were higher in clinical diagnoses but did not reach statistical significance (*p* > 0.05), while “m” and “ms” scores were consistent between methods across all age groups.

**TABLE 1:** The dmft and dmfs scores (mean ± standard deviation) in teledentistry and clinical diagnosis.

Age groups (year)	“dmft” scores		“dmfs” scores	
	Teledentistry diagnosis	Clinical diagnosis	Teledentistry diagnosis	Clinical diagnosis
	(Mean ± SD)	(Mean ± SD)	(Mean ± SD)	(Mean ± SD)
3–4	5.00 ± 3.51	5.93 ± 4.09	9.43 ± 7.81	10.93 ± 8.45
5	7.30 ± 3.40	7.85 ± 3.56	14.80 ± 8.88	15.90 ± 9.23
6	5.70 ± 4.46	6.37 ± 4.68	13.81 ± 13.12	15.04 ± 13.36
7	5.29 ± 2.78	6.46 ± 4.37	11.43 ± 7.16	13.40 ± 7.57
8	5.24 ± 3.17	5.60 ± 3.20	14.16 ± 9.87	15.00 ± 9.97
9	5.00 ± 2.59	5.38 ± 2.72	12.24 ± 9.35	13.38 ± 9.05
10	3.19 ± 2.46	3.37 ± 2.63	7.94 ± 5.85	8.31 ± 6.19
11–13	1.75 ± 1.96	1.75 ± 1.96	4.17 ± 5.72	4.25 ± 5.93

Abbreviation: SD, standard deviation.

**TABLE 2:** The d/m/f scores (mean ± standard deviation) in teledentistry and clinical diagnosis.

Age groups (year)	“d” scores		“m” scores		“f” scores	
	Teledentistry diagnosis	Clinical diagnosis	Teledentistry diagnosis	Clinical diagnosis	Teledentistry diagnosis	Clinical diagnosis
	(Mean ± SD)	(Mean ± SD)	(Mean ± SD)	(Mean ± SD)	(Mean ± SD)	(Mean ± SD)
3–4	4.79 ± 3.60	5.00 ± 4.54	0.00 ± 0.00	0.00 ± 0.00	0.21 ± 0.80	0.36 ± 1.34
5	5.85 ± 3.59	6.25 ± 3.88	0.50 ± 1.28	0.50 ± 1.28	0.95 ± 1.50	1.05 ± 1.50
6	4.37 ± 4.24	5.00 ± 4.31	0.15 ± 0.36	0.15 ± 0.36	1.19 ± 1.82	1.22 ± 1.97
7	4.29 ± 2.91	4.83 ± 3.03	0.23 ± 0.49	0.23 ± 0.49	0.77 ± 1.35	0.91 ± 1.58
8	3.92 ± 3.07	4.76 ± 4.82	0.52 ± 0.92	0.52 ± 0.92	0.80 ± 1.19	0.84 ± 1.25
9	4.07 ± 2.55	4.31 ± 2.55	0.28 ± 0.46	0.28 ± 0.46	0.66 ± 1.17	0.66 ± 1.17
10	2.50 ± 2.53	2.50 ± 2.56	0.13 ± 0.34	0.13 ± 0.34	0.63 ± 0.96	0.75 ± 1.18
11–13	1.67 ± 2.02	1.67 ± 2.02	0.00 ± 0.00	0.00 ± 0.00	0.17 ± 0.39	0.17 ± 0.39

Abbreviation: SD, standard deviation.

**TABLE 3:** The ds/ms/fs scores (mean ± standard deviation) in teledentistry and clinical diagnosis.

	“ds” scores		“ms” scores		“fs” scores	
Age groups (year)	Teledentistry diagnosis	Clinical diagnosis	Teledentistry diagnosis	Clinical diagnosis	Teledentistry diagnosis	Clinical diagnosis
	(Mean ± SD)	(Mean ± SD)	(Mean ± SD)	(Mean ± SD)	(Mean ± SD)	(Mean ± SD)
3–4	9.00 ± 7.94	10.07 ± 8.72	0.00 ± 0.00	0.00 ± 0.00	0.57 ± 2.14	0.86 ± 3.21
5	10.60 ± 8.49	11.35 ± 9.01	2.10 ± 5.25	2.10 ± 5.25	2.10 ± 3.13	2.40 ± 3.30
6	10.04 ± 12.59	11.00 ± 12.61	0.74 ± 1.81	0.74 ± 1.81	3.04 ± 4.25	3.30 ± 5.09
7	8.97 ± 7.33	10.11 ± 7.61	1.14 ± 2.45	1.14 ± 2.45	1.80 ± 2.76	2.14 ± 3.25
8	9.32 ± 9.18	9.48 ± 9.61	2.48 ± 4.28	2.48 ± 4.28	2.80 ± 5.31	2.04 ± 3.10
9	9.69 ± 9.14	10.52 ± 8.98	1.38 ± 2.27	1.38 ± 2.27	1.31 ± 2.57	1.48 ± 2.75
10	5.88 ± 6.12	6.06 ± 6.33	0.63 ± 1.71	0.63 ± 1.71	1.44 ± 2.13	1.63 ± 2.31
11–13	4.00 ± 5.58	4.08 ± 5.78	0.00 ± 0.00	0.00 ± 0.00	0.17 ± 0.39	0.17 ± 0.39

Abbreviation: SD, standard deviation.

Mean DMFT and DMFS values are outlined in Table 4. Clinical diagnosis yielded higher DMFT scores than teledentistry in all age groups except those aged 5–7, where both methods produced identical DMFT and DMFS values (*p* > 0.05). The DMFS scores were higher in clinical diagnoses across age groups, reaching statistical significance for the 11–13 age cohort (*p* = 0.02). Mean scores for additional parameters (“D, M, F, DS, MS, and FS”) are shown in Tables 5 and 6, with “D, F, DS, FS” scores being slightly higher in clinical assessments; however, these differences were not statistically significant (*p* > 0.05). Missing teeth assessments yielded similar results between the two diagnostic methods (*p* > 0.05).

**TABLE 4:** The DMFT and DMFS scores (mean ± standard deviation) in teledentistry and clinical diagnosis.

Age groups (year)	“DMFT” scores		“DMFS” scores	
	Teledentistry diagnosis (mean ± SD)	Clinical diagnosis (mean ± SD)	Teledentistry diagnosis (mean ± SD)	Clinical diagnosis (mean ± SD)
3–4	—	—	—	—
5	0.67 ± 1.16	0.67 ± 1.16	0.67 ± 1.16	0.67 ± 1.16
6	0.00 ± 0.00	0.00 ± 0.00	0.00 ± 0.00	0.00 ± 0.00
7	0.39 ± 0.70	0.39 ± 0.79	0.39 ± 0.70	0.39 ± 0.79
8	0.52 ± 0.92	0.68 ± 1.18	0.52 ± 0.92	0.76 ± 1.39
9	1.10 ± 1.40	1.30 ± 1.60	2.07 ± 3.43	2.40 ± 3.80
10	1.79 ± 1.84	2.16 ± 2.12	3.79 ± 5.59	4.32 ± 5.86
11–13	3.13 ± 2.33	4.00 ± 3.24	5.40 ± 4.60	7.07 ± 5.88

Abbreviation: SD, standard deviation.

**TABLE 5:** The D/M/F scores (Mean ± Standard Deviation) in teledentistry and clinical diagnosis.

Age groups (year)	“D” scores		“M” scores		“F” scores	
	Teledentistry diagnosis	Clinical diagnosis	Teledentistry diagnosis	Clinical diagnosis	Teledentistry diagnosis	Clinical diagnosis
	(Mean ± SD)	(Mean ± SD)	(Mean ± SD)	(Mean ± SD)	(Mean ± SD)	(Mean ± SD)
3–4	—	—	—	—	—	—
5	0.67 ± 1.16	0.67 ± 1.56	0.00 ± 0.00	0.00 ± 0.00	0.00 ± 0.00	0.00 ± 0.00
6	0.00 ± 0.00	0.00 ± 0.00	0.00 ± 0.00	0.00 ± 0.00	0.00 ± 0.00	0.00 ± 0.00
7	0.39 ± 0.70	0.36 ± 0.74	0.00 ± 0.00	0.00 ± 0.00	0.00 ± 0.00	0.03 ± 0.17
8	0.46 ± 0.78	0.56 ± 0.96	0.00 ± 0.00	0.00 ± 0.00	0.08 ± 0.40	0.12 ± 0.60
9	0.87 ± 1.22	1.10 ± 1.52	0.00 ± 0.00	0.00 ± 0.00	0.23 ± 0.68	0.23 ± 0.68
10	1.68 ± 1.83	1.89 ± 2.05	0.00 ± 0.00	0.00 ± 0.00	0.11 ± 0.32	0.26 ± 0.45
11–13	2.13 ± 1.66	2.53 ± 1.98	0.03 ± 0.18	0.07 ± 0.25	0.97 ± 1.40	1.43 ± 2.32

Abbreviation: SD, standard deviation.

**TABLE 6:** The DS/MS/FS scores (mean ± standard deviation) in teledentistry and clinical diagnosis.

Age groups (year)	“DS” scores		“MS” scores		“FS” scores	
	Teledentistry diagnosis	Clinical diagnosis	Teledentistry diagnosis	Clinical diagnosis	Teledentistry diagnosis	Clinical diagnosis
	(Mean ± SD)	(Mean ± SD)	(Mean ± SD)	(Mean ± SD)	(Mean ± SD)	(Mean ± SD)
3–4	—	—	—	—	—	—
5	0.67 ± 1.56	0.67 ± 1.16	0.00 ± 0.00	0.00 ± 0.00	0.00 ± 0.00	0.00 ± 0.00
6	0.00 ± 0.00	0.00 ± 0.00	0.00 ± 0.00	0.00 ± 0.00	0.00 ± 0.00	0.00 ± 0.00
7	0.39 ± 0.70	0.36 ± 0.74	0.00 ± 0.00	0.00 ± 0.00	0.00 ± 0.00	0.03 ± 0.17
8	0.44 ± 0.77	0.60 ± 1.08	0.00 ± 0.00	0.00 ± 0.00	0.08 ± 0.40	0.16 ± 0.80
9	1.30 ± 2.38	1.60 ± 2.90	0.00 ± 0.00	0.00 ± 0.00	0.77 ± 2.66	0.80 ± 2.68
10	3.63 ± 5.57	3.95 ± 5.86	0.00 ± 0.00	0.00 ± 0.00	0.16 ± 0.50	0.37 ± 0.68
11–13	3.17 ± 2.88	3.57 ± 3.12	0.17 ± 0.91	0.30 ± 1.15	2.07 ± 2.88	3.20 ± 4.71

Abbreviation: SD, standard deviation.

### 3.3 Prevalence of Dental Anomalies

Comparable prevalence rates were noted between teledentistry and clinical in‐person diagnoses for molar incisor hypomineralization (MIH) at 19%, black tooth stain at 5.5%, periodontal disease (unhealthy gingiva) at 28.5%, dental trauma at 5%, and orthodontic anomalies at 53%.

### 3.4 Treatment Guidance and Diagnostic Accuracy

Based on teledentistry evaluations, 28% of patients were recommended for preventive care, 8% for emergency interventions, and 64% for routine treatments. Medication (e.g., analgesics, antibiotics) was prescribed to 16% (*n* = 32) of patients presenting symptoms such as pain or swelling. Teledentistry demonstrated an 81% diagnostic accuracy rate for treatment needs. In clinical in‐person diagnosis, which incorporated radiographic assessments, several cases initially marked for restorative treatment were redirected to alternative interventions, such as vital pulp therapy, root canal procedures, or extractions.

### 3.5 Reliability Assessment

Repeated teledentistry and clinical in‐person evaluations conducted 1 week later on a random subset of 20 patients (10% of the cohort) demonstrated no statistically significant variations across parameters (*p* > 0.05). The intraclass correlation coefficient (ICC) for repeated measures was calculated as 0.91, reflecting high reliability.

## 4 Discussion

In recent decades, technological advancement has significantly accelerated, especially within telecommunications (e.g., smartphones, computers), software applications, and connectivity options (e.g., cellular networks, wi‐fi). These advancements have catalyzed the development of telemedicine devices that support remote healthcare delivery. Smartphones' widespread adoption has facilitated access to healthcare services directly via personal mobile devices. Teledentistry offers distinct advantages, such as minimizing patient clinical visits and reducing chair time. Virtual consultations enable dentists to manage a higher volume of patients daily and to readily consult specialists for second opinions, provided patient consent is obtained. This approach is especially advantageous for patients in geographically remote areas, as it reduces travel needs and shortens waiting times in dental offices. Additionally, teledentistry presents a cost‐effective alternative to in‐person dental care without compromising quality where clinically appropriate. While prior research on teledentistry has primarily focused on adult populations or specific dental conditions, a notable gap remains in comprehensive studies evaluating its reliability across a broad spectrum of pediatric dental issues [3, 4, 14]. This study aims to address that gap by providing a direct comparison between teledentistry and conventional clinical diagnosis specifically in pediatric patients, who present distinct behavioral and diagnostic challenges.

Telemedicine applications provide timely access to dental examinations, allowing urgent cases to receive initial assessments before referral to a healthcare institution in remote settings [3, 4]. Although teledentistry, a subset of telemedicine, began in the 1990s, it remains a relatively new concept with considerable potential to transform global oral healthcare delivery [17]. Teledentistry applications were initially developed to reduce treatment costs and improve patient care by streamlining referral processes, departing from conventional methods [1, 2].

The COVID‐19 pandemic further accelerated the adoption of telehealth, including teledentistry, underscoring its potential to expand care access and enhance patient outcomes. In urgent cases, patients can remotely contact their dentist, who can assess the situation before prescribing medication, ultimately conserving resources and reducing unnecessary clinical visits. The literature suggests that teledentistry can be effective with noncooperative pediatric patients. However, our study did not reflect this finding. Our inclusion criteria necessitated clear photographic and video submissions, inherently favoring patients who were already cooperative. Consequently, all patients who attended clinical examinations displayed compliant behavior (rated as 3 (positive) or 4 (definitely positive) on the Frankl Behavior Scale). Future studies might address this limitation by assessing the effectiveness of teledentistry with pediatric patients across different compliance levels, using standardized behavioral scales for more diverse group selection.

McLaren et al. reported that 88% of dental treatment plans determined via teleconsultation remained consistent with clinical diagnoses, supporting the effectiveness of teleconsultation for pediatric patients as well [18]. This study investigated the diagnostic reliability of teledentistry in 200 children aged 3–13 during the COVID‐19 pandemic, comparing teledentistry to traditional clinical dental diagnosis. Consistent with previous findings, data from this study demonstrated compatibility between teledentistry and in‐person diagnostic methods.

In capturing dental images through smartphone cameras, it is essential that the individual transmitting the image aims for optimal quality to support accurate diagnosis in teledentistry. Insufficient focus or lighting, especially at tooth interfaces, may hinder detection of caries or restorations through teledentistry alone [19].

AlShaya et al. assessed teledentistry for caries detection in pediatric patients, where intraoral and extraoral images captured via mobile cameras were securely stored and transmitted to pediatric dentists through social media platforms (WhatsApp, Messenger, etc.). Although the absence of radiographs in teledentistry reduces diagnostic accuracy compared to clinical examinations, the authors concluded that mobile‐based teledentistry offers reliable preliminary caries detection in children [20]. Similarly, Telles‐Araujo et al. highlighted the utility of WhatsApp for teledentistry, emphasizing the importance of high‐resolution images for accurate diagnoses [21].

In our country, WhatsApp is widely utilized for instant messaging, video calls, and image transmission without extra charges, making it an ideal choice for teledentistry diagnostics in this study. WhatsApp's end‐to‐end encryption also ensures secure data sharing. In our study, all images were securely stored on Google Drive, a password‐protected, encrypted platform that maintains data privacy until explicitly shared with authorized individuals. Despite the challenges posed by data security, regulatory, and licensing issues in teledentistry, the applications used in this study (WhatsApp, Google Drive) are secure, widely accepted platforms that support confidentiality in data transmission and storage.

In this study, we employed a Caries Risk Assessment Tool (CAT) [15] and the World Health Organization (WHO) caries recording indices (DMFT/dmft, DMFS/dmfs) to assess caries risk and caries prevalence. The CAT is a validated instrument designed to evaluate the likelihood of dental caries development based on various clinical and behavioral factors. Its validation has been rigorously conducted through extensive research, demonstrating its reliability and predictive accuracy in diverse populations. No artificial intelligence‐supported tools specific to teledentistry were used. The teledentistry diagnosis was performed through the ‘store and forward’ method, utilizing smartphones and the WhatsApp application for photo and video sharing. Results indicated that 77% of the 200 patients were categorized as high caries risk according to CAT. Caries index scores were evaluated across all age groups, revealing a high level of agreement between teledentistry and clinical diagnostic scores. This aligns with findings by Estai et al., who demonstrated that smartphone imaging and cloud‐based platforms can serve as reliable alternatives to clinical examinations for oral disease diagnosis [22, 23].

Upon comparing “dmft, dmfs, DMFT, and DMFS” scores in high‐risk patients, challenges arose in identifying the exact number of carious teeth and surfaces, particularly in younger children with high scores. Periodontal issues and food retention further complicated carious surface detection. The reduced accuracy in posterior area may stem from several inherent limitations in remote diagnostic imaging. First, imaging of posterior teeth often requires precise angulation, adequate lighting, and intraoral access, conditions that can be challenging to achieve with smartphone cameras and in‐home settings. In teledentistry, suboptimal image quality and insufficient focus are common issues that can obscure the visibility of carious lesions, particularly at interproximal surfaces where early‐stage caries are most likely to occur and are thus harder to detect without professional dental lighting and equipment. Additionally, younger pediatric patients tend to have limited mouth opening and movement control, complicating image capture of posterior regions and affecting diagnostic accuracy. It is recommended that parents brush the child's teeth before image capture to improve diagnostic accuracy in the present study. Challenges were also encountered in detecting resin‐based restorations due to their advanced color and surface esthetics, whereas glass ionomer restorations, with their opaque structure, were more readily identifiable. This observation contrasts with findings by Pentapati et al. [24], where a trained dental assistant utilized an intraoral camera for image capture, indicating potential methodological differences. In this study, we observed a high concordance between teledentistry and clinical in‐person dental diagnosis for anterior caries detection, which supports findings by Estai et al. [22, 23], who demonstrated that smartphone imaging can be a reliable diagnostic tool for detecting visible oral diseases. However, the present study diverges from Estai's findings in posterior caries detection, where accuracy was notably lower. This discrepancy may result from the technical limitations of mobile imaging, particularly in capturing high‐quality images of posterior teeth, as well as the challenges posed by younger pediatric patients with limited cooperation, factors less emphasized in Estai's work. Moreover, while AlShaya et al. [20] reported acceptable diagnostic reliability for initial caries detection via teledentistry, our findings suggest that certain carious lesions, especially those in posterior regions or on interproximal surfaces, may not be consistently detected without radiographic support, highlighting an area for improvement in teledentistry's diagnostic protocols. Future integration of specialized intraoral cameras and artificial intelligence (AI) algorithms trained to detect posterior caries in teledentistry could address these limitations, potentially narrowing the accuracy gap observed between remote and clinical diagnostic methods.

A detailed analysis of each tooth demonstrated similar diagnostic accuracy between teledentistry and clinical examination for missing teeth (*p* > 0.05). Caries (D/d) and filling (F/f) detection were more accurate in the anterior region via teledentistry; however, diagnoses in the posterior region, particularly the maxillary posterior, proved challenging. Children with rampant caries and those younger than 5 years often presented with limited mouth opening, leading to suboptimal imaging and 19% of diagnoses being missed by teledentistry in comparison to clinical diagnosis.

To evaluate measurement reliability, the intraclass correlation coefficient (ICC) was calculated for repeated assessments by the same observer in both teledentistry and clinical settings. Given the high‐quality images obtained from cooperative pediatric patients, the ICC was notably high (0.91), underscoring robust reliability and diagnostic concordance between the two modalities.

In this study, comparisons extended beyond caries index status to include various dental conditions such as molar incisor hypomineralization, black tooth staining, periodontal health (healthy/unhealthy gingiva), dental trauma, and orthodontic anomalies. Findings indicated a similar prevalence of these anomalies across both teledentistry and clinical diagnoses, aligning with the results reported by Damoiselet et al. [25].

As part of the broader telehealth framework, teledentistry has rapidly expanded in response to advancements in information and communication technologies and reductions in related costs [5, 6, 26]. Existing studies on teledentistry have largely focused on promoting oral hygiene, identifying dental anomalies, and managing patient care during orthodontic treatment [12, 20, 26]. However, research specifically assessing the effectiveness and reliability of teledentistry in pediatric populations remains limited. To our knowledge, no study has comprehensively compared teledentistry with clinical diagnoses across a range of dental issues—such as dental caries, periodontal health, molar incisor hypomineralization, black tooth staining, dental trauma, orthodontic anomalies, and treatment recommendations—in the pediatric context.

Recent developments have introduced artificial intelligence (AI)‐integrated telehealth applications, such as AICaries, DentalAI, and SMARTeeth, which are compatible with smartphones and designed to aid in the early detection and management of oral health issues. A limitation of the present study is the absence of these advanced AI‐compatible applications in the teledentistry diagnostic process. Future research would benefit from the incorporation of such tools to enhance diagnostic capabilities.

In conclusion, teledentistry has proven its value in facilitating dental care delivery, particularly during the COVID‐19 pandemic, by providing safe, accessible, and effective remote consultation options that maintain the continuity of oral healthcare. As digital technologies advance, the integration of teledentistry into routine dental practice presents significant potential for the future of oral healthcare. For pediatric dentistry, teledentistry can serve as a valuable supplementary tool, especially in emergency scenarios or remote areas where initial assessments may otherwise be delayed. Our findings contribute to the literature by establishing teledentistry as a viable diagnostic tool across various pediatric dental conditions, addressing a critical need for accessible, efficient, and reliable dental care options for this vulnerable age group. The present study demonstrates that teledentistry can serve as a reliable diagnostic tool for pediatric dental care, particularly for conditions such as caries, periodontal health and other dental problems. To enhance the reliability of teledentistry in practice, clinicians could implement standardized protocols for image capture that emphasize optimal lighting, angulation, and patient positioning, thereby improving the quality of transmitted images. Additionally, integrating advanced diagnostic technologies, such as AI algorithms capable of analyzing captured images for carious lesions, could significantly enhance diagnostic accuracy, especially in posterior regions where detection challenges persist. The broader implications of these findings extend to improving access to dental care in underserved areas, where clinical in‐person visits may be limited by geographic or economic barriers. By leveraging teledentistry, dental practitioners can provide timely consultations and treatment recommendations, ensuring that vulnerable populations receive essential dental care despite these challenges. Ultimately, the integration of teledentistry into routine pediatric dental practice not only facilitates immediate access to care but also promotes preventive measures, contributing to improved oral health outcomes in communities that may otherwise face significant barriers to treatment.

## Author Contributions

Müge Erbay Mola and Dilşah Çoğulu conceived the ideas, designed the project, and wrote the manuscript. Müge Erbay Mola also collected and analyzed the data. Ece Eden and Aslı Topaloğlu wrote and revised the manuscript. All authors read and approved the final manuscript.

## Ethics Statement

The Ethical Committee of the Medical Faculty of Ege University approved the study (Reference No: 21‐2.1T/62).

## Consent

All participants were comprehensively informed of the research objectives, and detailed information about the treatment regimen was provided to both the children and their parents. Written informed consent was obtained, ensuring all individuals; voluntary and consensual participation. Data will be used exclusively for research purposes, maintaining strict confidentiality and privacy.

## Conflicts of Interest

The authors declare no conflicts of interests.

## Data Availability

The results data for this research are exclusively accessible within the article and is not available on any external platform due to publication constraints. Interested parties may request access to the results dataset by contacting the authors directly through the provided contact information in the article.
